# Astrocyte depletion alters extracellular matrix composition in the demyelinating phase of Theiler’s murine encephalomyelitis

**DOI:** 10.1371/journal.pone.0270239

**Published:** 2022-06-17

**Authors:** Lisa Allnoch, Eva Leitzen, Isabel Zdora, Wolfgang Baumgärtner, Florian Hansmann

**Affiliations:** 1 Department of Pathology, University of Veterinary Medicine Hannover, Hannover, Germany; 2 Center for Systems Neuroscience, Hannover, Germany; 3 Institute for Veterinary Pathology, Veterinary Faculty, Leipzig University, Leipzig, Germany; University of Crete, GREECE

## Abstract

Astrocytes produce extracellular matrix (ECM) glycoproteins contributing to the blood-brain barrier and regulating the immune response in the central nervous system (CNS). The aim of this study was to investigate the impact of astrocyte depletion upon the clinical outcome and the composition of ECM glycoproteins in a virus-induced animal model of demyelination. Glial fibrillary acidic protein (GFAP)-thymidine-kinase transgenic SJL (GFAP-knockout) and wildtype mice were infected with Theiler’s murine encephalomyelitis virus (TMEV). Astrocyte depletion was induced during the progressive, demyelinating disease phase by ganciclovir administration once daily between 56 and 77 days post infection (dpi). At 77 dpi GFAP-knockout mice showed a significant deterioration of clinical signs associated with a reduction of azan and picrosirius red stained ECM-molecules in the thoracic spinal cord. Basement-membrane-associated ECM-molecules including laminin, entactin/nidogen-1 and Kir4.1 as well as non-basement membrane-associated ECM-molecules like collagen I, decorin, tenascin-R and CD44 were significantly reduced in the spinal cord of GFAP-knockout mice. The reduction of the investigated ECM-molecules demonstrates that astrocytes play a key role in the production of ECM-molecules. The present findings indicate that the detected loss of Kir4.1 and CD44 as well as the disruption of the integrity of perineuronal nets led to the deterioration of clinical signs in GFAP-knockout mice.

## Introduction

Astrocytes play an important role in diseases of man such as multiple sclerosis (MS) as well as in animals like canine distemper encephalitis and Theiler’s murine encephalomyelitis virus-induced demyelinating disease (TMEV-IDD) [1–5]. TMEV-IDD is a widely used animal model for the investigation of specific aspects regarding the pathogenesis of the progressive forms of MS [6–8]. Hallmarks of TMEV-IDD include spinal cord inflammation and demyelination which can be observed in white matter lesions during the chronic phase of TMEV-IDD together with axonal degeneration and loss [6, 9]. Different hypotheses regarding the development of demyelination during TMEV-IDD include the inside-out model assuming a primary axonal damage followed by demyelination as well as the outside-in model postulating a primary demyelination with subsequent axonal damage [10].

Astrocytes express multiple pattern recognition receptors (PRRs) and can mediate the innate immune response by directly regulating the entry of inflammatory cells through the blood-brain barrier (BBB) / blood-spinal cord barrier (BSCB) as well as by secretion of various cytokines including MCP-1, RANTES, CXCL10, CXCL12, and IL-8, all attracting peripheral immune cells and microglia [11, 12]. Following injury, astrocytes form glial scars leading to an impairment of remyelination and axonal regeneration [11, 13]. Furthermore, astrocytes are critically involved in the maintenance of the highly dynamic CNS microenvironment [14–16]. In order to preserve the neuroprotective milieu in both physiologic and inflamed CNS, astrocytes secrete numerous extracellular matrix (ECM) glycoproteins including laminins, secreted protein acidic and rich in cysteine (SPARC), tenascin-C, and thrombospondin [17]. The ECM itself is necessary for cell trafficking, tissue remodeling and determines numerous biological processes including BBB integrity, synapse formation and maintenance, regulation of the ionic and nutritional homeostasis as well as de- and remyelination [18–23].

**Fig 1:**
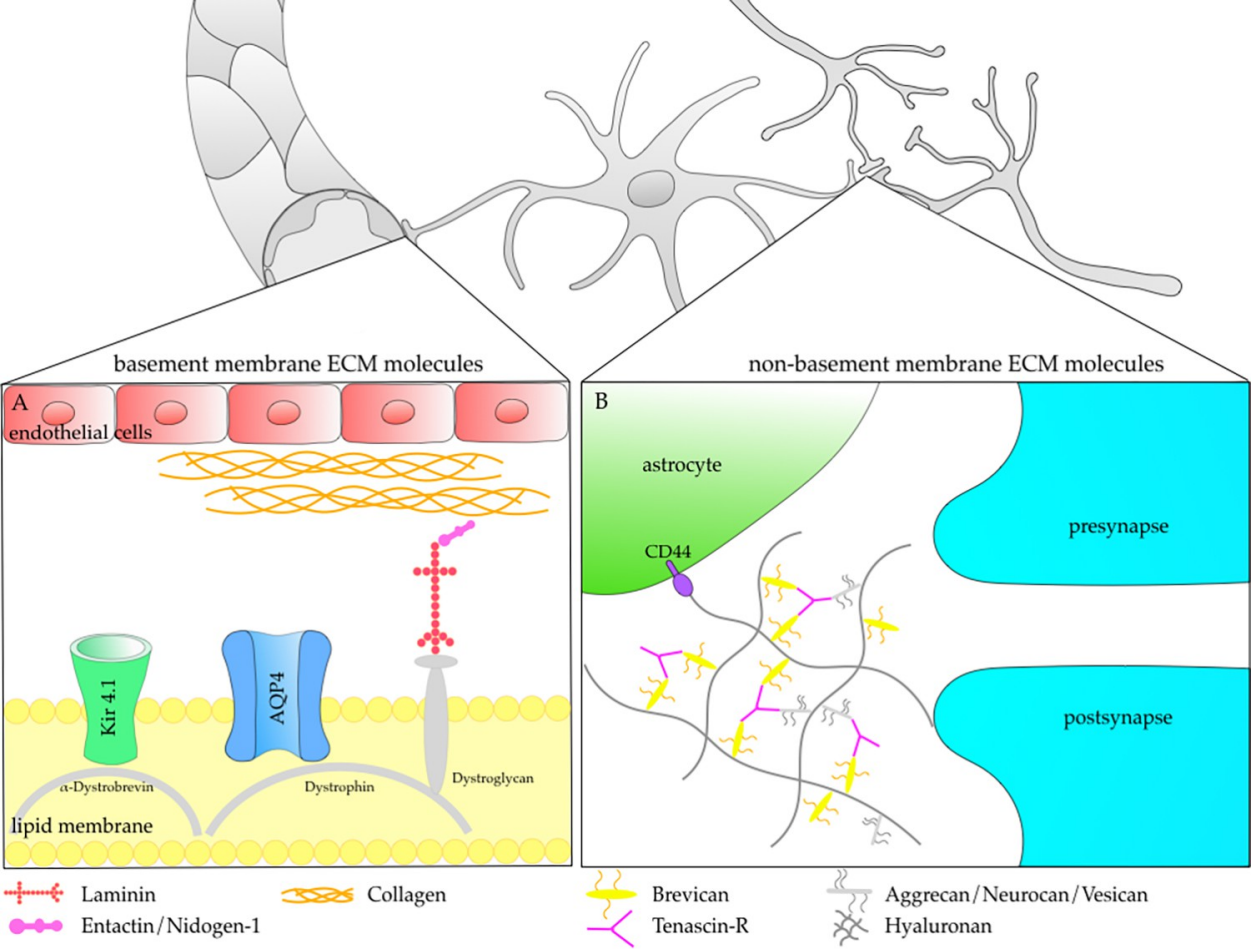
Localization and function of basement membrane and non-basement membrane extracellular matrix (ECM) molecules. Detailed view (**A**) shows basement membrane ECM molecules like laminin, entactin/nidogen-1 and collagen. Laminin interacts within the dystroglycan-dystrophin-dystrobrevin complex to ensure anchoring and stabilization of the aquaporin 4 (AQP4) water channel as well as the inwardly rectifying potassium channel Kir 4.1 in the perivascular basal lamina [32, 33]. Detailed view (**B**) illustrates the participation of non-basement membrane ECM molecules like brevican and tenascin-R in the tripartite synapse. The tripartite synapse consists of a presynaptic and postsynaptic membrane as well as a closely apposed astrocyte process [44]. By connection to further ECM components (hyaluronan, aggrecan, neurocan, vesican) tenascin-R and brevican form perineuronal nets (PNN). Anchored via the cell-surface glycoprotein CD44, PNNs are presumed to play a role in synapse stability and plasticity [44].

Astrocyte derived ECM glycoproteins belong to the group of basement and non-basement molecules (Fig 1) [13]. The ECM molecule laminin plays an important role in the basement membrane of the BBB (Fig 1A) [15, 24, 25]. In response to CNS injury, laminin is quickly upregulated in astrocytes and endothelial cells [15, 26, 27]. At present, 16 laminin isoforms with different distribution pattern in combination with a specific, temporal and spatial regulation are described [15, 28]. Several of these isoforms, including α1–5, β1, and γ1 were identified in the rodent brain [15, 29, 30]. The α2 isoform of laminin is located in the basement membrane of cerebral blood vessels and may contribute to the selective filtration of the BBB [15, 31]. Via the dystroglycan-dystrophin-dystrobrevin complex, laminin is coupled to further molecules of the lipid membrane, such as the water channel aquaporin 4 (AQP4) and the inwardly rectifying potassium channel Kir 4.1, two channel proteins involved in preservation of CNS homeostastis [32, 33]. After neuronal excitation, Kir 4.1 channels release potassium into the extracellular space and thus regulate neuronal excitability and ensure a neuroprotective milieu [34]. Generating Kir 4.1 knockout mice resulted in animals exhibiting severe motor impairment including ataxia, tremor, stress-induced seizures, white-matter vacuolization and early mortality [35, 36].

Non-basement membrane ECM molecules include, among others, collagen I, decorin, tenascin-R and brevican (Fig 1B) [13]. Tenascin-R is involved in CNS repair and plasticity by activating microglia to induce secretion of growth factors and cytokines including brain-derived neurotrophic factor (BDNF), nerve growth factor (NGF), and transforming growth factor-β (TGF-β) [34]. In addition, tenascin-C, tenascin-R, neurocan, and brevican have a significant impact upon the ratio of excitatory to inhibitory synapses and thereby modulate tripartide synapse formation and remodeling [35]. Non-basement membrane ECM molecules such as tenascin-R and brevican are in particular anchored to CD44 at astrocytic endfeet using a hyaluronan backbone [36, 37]. CD44 belongs to a family of transmembrane glycoproteins and is critically involved in ECM remodeling, cellular adhesion, and hyaluronan degradation [38, 39]. In the central nervous system, CD44 is not only produced by astrocytes but also by neurons and oligodendrocyte progenitor cells [OPCs; 40]. Within the lesioned CNS an accumulation of CD44 in the CNS parenchyma is reported [41–43].

In a previous study, we used *SJL*.*Cg-Tg(Gfap-TK)*^*7*.*1Mvs*^ (GFAP knockout) mice to investigate the effects of astrocyte depletion upon inflammation, demyelination, and axonal damage during TMEV-IDD [4]. TME virus (TMEV) infected GFAP knockout mice showed a significant reduction of inflammation in the thoracic spinal cord compared to wildtype mice, while the severity of axonal damage and demyelination was unaltered [4]. Surprisingly, despite exhibiting less inflammation, astrocyte depleted animals still showed a significant deterioration of clinical signs as determined by a clinical scoring system and a rotarod performance test [4]. Immunohistochemical and ultrastructural investigations confirmed a significant reduction of astrocytes [4] and revealed a reduced and disorganized expression of AQP4 water channels in astrocytic foot processes of TMEV infected GFAP knockout mice [4]. The aim of the present study was to further detail the underlying associated mechanisms causing a worsening of clinical signs in GFAP knockout mice despite the fact that reduced inflammation did not change the degree of demyelination and axonal damage in affected mice. Therefore, the composition of astrocyte associated basement membrane and non-basement membrane ECM components and selected, associated molecules were investigated.

## Materials and methods

### Animal experiments

For animal experiments female, five to six-week-old *SJL*.*Cg-Tg(Gfap-TK)*^*7*.*1Mvs*^ and *SJL/JOlaHsd* mice were used [4]. Animals were housed in isolated ventilated cages (Tecniplast, Hohenpeißenberg, Germany) in a standardized environment. Animals had free access to water and were fed a standard rodent diet (R/M-H; Ssniff Spezialdiäten GmbH, Soest, Germany). Animal experiments were performed according to the German Animal Welfare Law, in compliance with the ARRIVE (Animal Research: Reporting of In Vivo Experiments) guidelines and approved by the local authorities (Lower Saxony State Office for Consumer Protection and Food Safety (LAVES), Oldenburg, Germany, permission number: 33.12-42502-04-12/0949).

### Intracerebral TMEV infection and ganciclovir treatment

Intracerebral injection of TMEV (1.14 × 10^5^ plaque-forming units per mouse, BeAn strain, passage 3) into the right cerebral hemisphere was performed under general anesthesia (100 mg/kg ketamine, Ketamin 10%, WDT, Garbsen, Germany and 0.5 mg/kg medetomidine, Domitor®, Pfizer, Berlin, Germany) as previously described [3]. Mice received an intraperitoneal injection of either ganciclovir (25 mg/kg, Cymevene™, Par Sterile Product LLC, United States) or sodium chloride solution (WDT, Garbsen, Germany) once daily from 56 to 77 days post TMEV infection [3, 4]. A total of 20 animals were randomly assigned into 4 treatment groups, namely TMEV infected, ganciclovir treated, GFAP-transgenic SJL mice (GSTG; n = 5), TMEV infected, NaCl treated, GFAP-transgenic SJL mice (GSTP; n = 4), TMEV infected, ganciclovir treated wildtype SJL mice (WSTG; n = 5), and TMEV infected, natrium chloride treated wildtype SJL mice (WSTP; n = 6). Random numbers were generated using the RAND() function in Microsoft Excel. The selected group size was based on previous experiments with TMEV-IDD in SJL mice considering mean and standard deviations regarding axonal damage and demyelination. Animals were included in the study following necropsy at the scheduled time point while 1 animal was excluded since it died prior to the scheduled time point.

### Clinical examination and tissue sampling

For clinical investigation, a scoring system evaluating the categories posture and physical appearance, behavior, activity as well as gait was applied [4]. Furthermore, motor coordination was evaluated using a rotarod (RotaRod treadmill, TSE Technical & Scientific Equipment, Bad Homburg, Germany), which was accelerated from 5 rounds per minute (rpm) to 55 rpm over a duration of five minutes [4]. Clinical evaluation was performed daily in randomized order between 8 a.m. to 8 p.m. Investigators could not be blinded to the treatment groups due to the fact that part of the animals were virus-infected. Mice were euthanized and transcardially perfused with phosphate buffered saline (PBS, pH 7.4) at 77 dpi. From all animals the thoracic (T3-5) spinal cord segment was fixed in 10% buffered formalin.

### Histochemistry

Formalin-fixed thoracic spinal cord samples were embedded in paraffin wax. 2–3-μm-thick transversal serial sections of the spinal cord were prepared and stained with a modified azan and a modified picrosirius red (PSR) stain for detection of ECM components [13, 45]. For the former, slides were deparaffinized and rehydrated. Sections were stained in 5% phosphotungstic acid for 15 minutes and rinsed in distilled water. Thereafter, slides were stained in aniline blue-orange G-acetic acid (0,5 g aniline blue and 2 g orange G in 100 ml distilled water supplemented by 8 ml acetic acid, boiled, filtrated and diluted 1:2 in distilled water) for 20 minutes. After repeated rinsing in distilled water sections were dipped in 96% ethanol followed by dehydration and mounting. This modified azan staining resulted in a blue color of acidic mucosubstances.

For PSR staining, slides were deparaffinized and rehydrated. After dipping in 3% acetic acid, sections were stained in 1% alcian blue solution pH 2.5 (1 g alcian blue 8GX in 100 ml 3% acetic acid supplemented by 1 crystal of thymol) for 30 minutes. Afterwards, slides were rinsed in tap water for 3 minutes and dipped in 70% ethanol for 15 seconds followed by staining in aldehyde fuchsin for 45 minutes. After dipping in 70% ethanol and rinsing in tap water for 3 minutes, slides were stained in Weigert’s hematoxylin (ferric chloride solution and 1% hematoxylin in absolute alcohol in equal amounts) for 10 minutes. Sections were rinsed again in tap water and stained in 0.1% PSR solution (0.1 g sirius red F3B combined with 100 ml saturated aqueous picric acid) for 60 minutes. Finally, slides were dipped in 0.01% hydrochloric acid, followed by dehydration and mounting. The modified PSR staining led to a red color of sulfated mucosubstances like proteoglycans and glycoproteins as well as collagens.

### Immunohistochemistry

Immunohistochemistry was performed as previously described [3, 7, 46, 47]. Briefly, sections were dewaxed by incubation for 5 minutes twice in Roticlear® (Roth C. GmbH & Co. KG, Karlsruhe, Germany) and rehydrated in isopropanol and ethanol (96%; each for 5 minutes). Subsequently, endogenous peroxidase activity was blocked by incubating the sections in 85% methanol (collagen I) or ethanol (all other antibodies) with 0.5% H_2_O_2_ for 30 minutes at room temperature. If required, antigen retrieval was performed by incubating slides for 20 minutes in boiling citrate buffer (pH 6) in a microwave (800W) or with 0.5% triton X-100 for 15 minutes (for brevican; Table 1). Blocking of nonspecific binding was performed incubating the slides for 20 minutes in normal goat serum or normal rabbit serum diluted 1:5 in PBS (pH 7.2). For visualization of antigen-antibody reactions the avidin–biotin-peroxidase complex (ABC) method (Vector Laboratories, Burlingame, CA, USA) and 3,3´-Diaminobenzidine tetrahydrochloride (Sigma-Aldrich, St. Louis, MO, United States) were used. Primary antibodies were diluted in PBS containing 1% bovine serum albumin and incubated overnight at 4°C. Applied antibodies, dilutions, and pretreatments are detailed in Table 1.

**Table 1:** Primary antibodies, pretreatment and dilutions used for immunohistochemistry.

Antigen	Pretreatment	Dilution	Clonality	Supplier	Catalog number
**Brevican**	0.5% triton	1:100	polyclonal rabbit	Cloud-Clone Corp, Katy, USA	A20191030263
**CD44**	microwave/citrate buffer	1:4000	polyclonal rabbit	Abcam, Cambridge, UK	ab15707
**Decorin**	/	1:200	polyclonal goat	R&D Systems, Abingdon, UK	AF1060
**Entactin / nidogen-1**	microwave/citrate buffer	1:3000	monoclonal rabbit	Abcam, Cambridge, UK	GR3276738-2
**Kir 4.1**	microwave/citrate buffer	1:6000	polyclonal rabbit	Alomone labs, Jerusalem, Israel	apc 035
**Collagen I**	microwave/citrate buffer	1:600	polyclonal rabbit	Abcam, Cambridge, UK	ab21286
**Laminin**	/	1:50	polyclonal rabbit	Sigma-Aldrich Chemie GmbH, Taufkirchen, Germany	L9393
**Tenascin-R**	microwave/citrate buffer	1:750	polyclonal rabbit	Boster Biological Technology Co., Ltd, California, USA,	PA1695-1

### Evaluation and statistical analysis

For quantifying the azan and PSR positive area as well as for brevican, CD44, decorin, entactin/nidogen-1, Kir 4.1, collagen I, laminin, and tenascin-R immunohistochemistry one picture per marker was taken of the thoracic spinal cord segment with a photo microscope (BZ-9000E, Keyence Deutschland GmbH, Neu-Isenburg, Germany). On every picture the ventral thoracic spinal cord white matter was outlined as region of interest. Positive area was detected and quantified by digital image analysis (analySIS®, Soft Imaging System GmbH, Münster, Germany). All sections were evaluated by one investigator (L.A.) blinded to the treatment conditions. Statistical analyses were conducted using IBM SPSS™ (version 21, New York, USA) for Windows™. Data analysis included Mann-Whitney U tests. Statistical significance was accepted at p-values of ≤ 0.05 (*) and ≤ 0.01 (**), respectively. Graphs were designed using GraphPad Prism (GraphPad Software, San Diego, CA, USA) for Windows™.

## Results

### Clinical findings and histological characterization of spinal cord lesions

**Fig 2:**
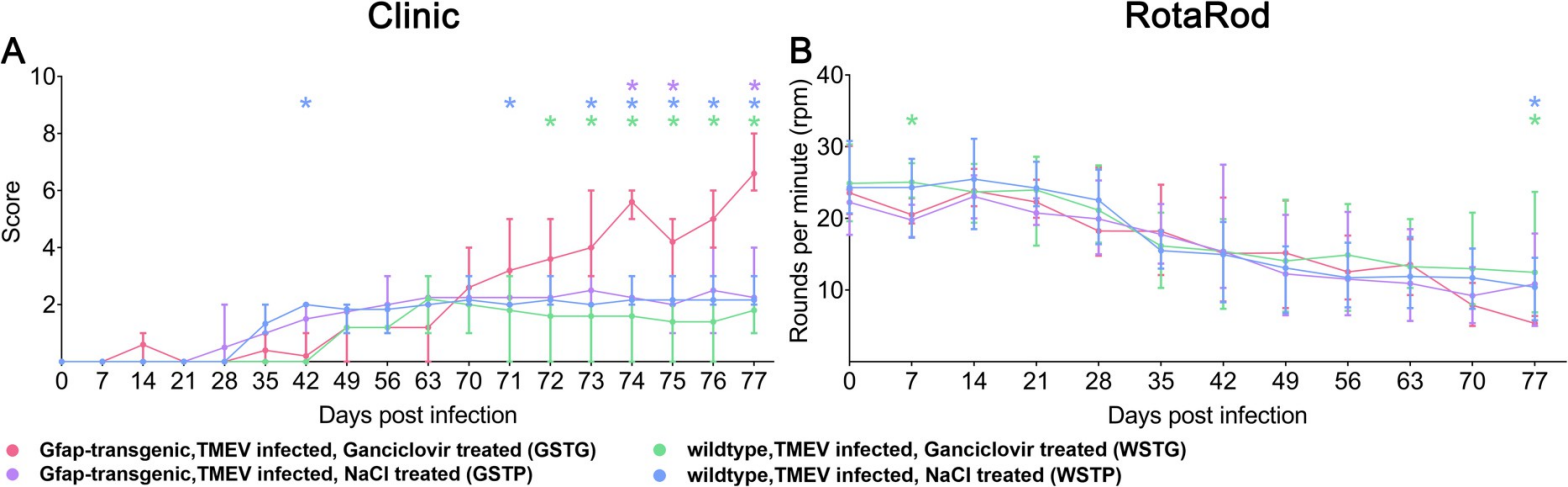
Clinical investigations. Astrocyte depletion between 56–77 days post infection (dpi) resulted in a deterioration of clinical signs starting at 71 dpi compared to TMEV infected, NaCl treated, GFAP-transgenic (GSTP) mice (purple asterisks), TMEV infected, ganciclovir treated wildtype (WSTG) mice (green asteriks) and TMEV infected, natrium chloride (NaCl) treated wildtype (WSTP) mice (blue asterisks, A). At 77 dpi TMEV infected, ganciclovir treated, GFAP-transgenic mice (GSTG) mice showed a significant detetioration of rotarod performance compared to the WSTG and WSTP controls (green and blue asterisks, B). Clinical and rotarod data are shown as mean and standard error of mean. Significant differences between GSTG and the respective control groups, as obtained by Kruskal–Wallis test, followed by Mann–Whitney U post hoc tests were indicated by *, p < 0.05. Part of these data were previously published [4].

TMEV infected, ganciclovir treated, GFAP-transgenic mice (GSTG) showed a significant deterioration of clinical signs including reduced general condition, shaggy fur, waddling gait, and progressive ataxia starting at 71 dpi ([4]; Fig 2A). Rotarod performance test identified a significant deterioration of motor coordination in GSTG mice at 77 dpi ([4]; Fig 2B).

### Effects of astrocyte depletion upon the extracellular matrix

**Fig 3:**
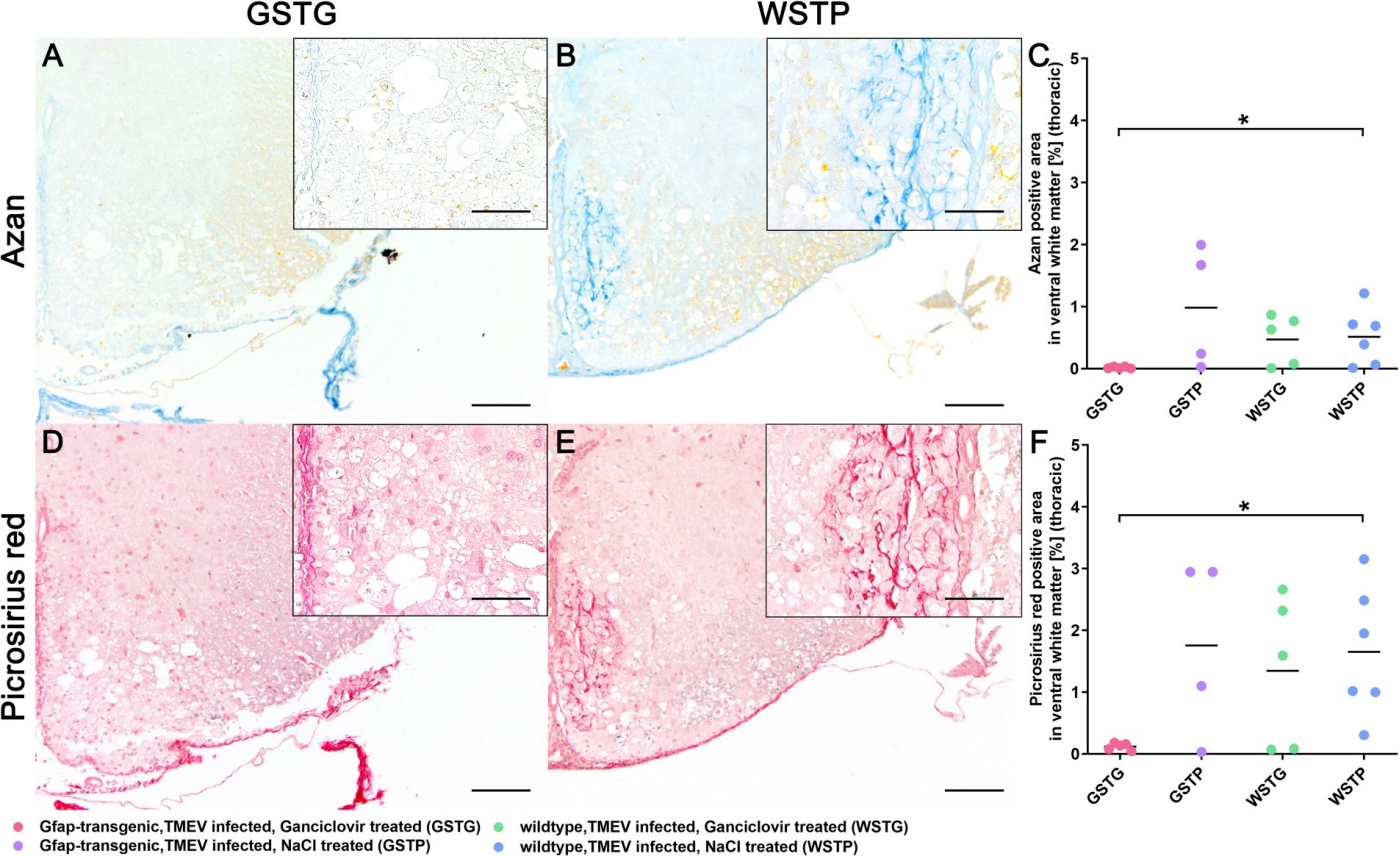
Impact of astrocyte depletion upon the deposition of extracellular matrix (ECM). Quantification of ECM components in the thoracic spinal cord white matter was performed using azan (**A-C**) and picrosirius red (**D-F**) staining. Accumulation of ECM components was detected intralesionally within the ventral part of the thoracic spinal cord white matter. At 77 dpi TMEV infected, ganciclovir treated, GFAP-transgenic mice (GSTG) mice (**A, D**) showed a significantly reduced azan and picrosirius red positive area, respectively, compared to TMEV infected, natrium chloride (NaCl) treated wildtype (WSTP) animals (**B, C, E, F**). Inserts visualize in more detail the intralesional accumulation of azan (**B**) and picrosirius red (**E**) labeled ECM molecules in WSTP animals compared to astrocyte depleted mice (**A**, **D**). For each antibody, one cross section of the thoracic spinal cord was evaluated per animal. Data are shown as scatter dot plots. The horizontal bar indicates the mean. Significant differences between GSTG and the control groups obtained by Kruskal-Wallis test followed by Mann–Whitney U post hoc tests are indicated by *, p ≤ 0.05. Bars represent 100 μm in overviews and 50 μm in the inserts.

ECM remodeling is a continuous process in the CNS of healthy and diseased individuals. Previous studies in TMEV-IDD showed an increased deposition of ECM components including laminin, entactin, collagen I, and decorin during the progression of TME, while in age matched controls a low amount of these ECM components was detected [13]. In the present study the impact of astrocyte depletion upon the amount of ECM accummulation during the chronic phase of TMEV-IDD was investigated using azan and PSR stainings (Fig 3). The blue color of the azan staining indicates acidic mucosubstances (Fig 3A and 3B), while the red color in the PSR staining visualizes sulfated mucosubstances like proteoglycans and glycoproteins as well as collagens (Fig 3D and 3E). Azan and PSR positive material was detected with variable degree accentuated in the lesioned ventral part of the thoracic spinal cord white matter in all TMEV infected groups. At 77 dpi a significant reduction of the azan and picrosirius red positive area was detected in GSTG compared to WSTP mice (Fig 3C and 3F).

### Impact of astrocyte depletion upon basement membrane ECM molecules

Since earlier studies postulated a correlation between the loss of astrocyte morphology and ECM composition, immunohistochemistry targeting astrocyte associated basement-membrane ECM molecules including laminin and entactin/nidogen-1 was performed to specify the results of the histochemical investigations [4].

**Fig 4:**
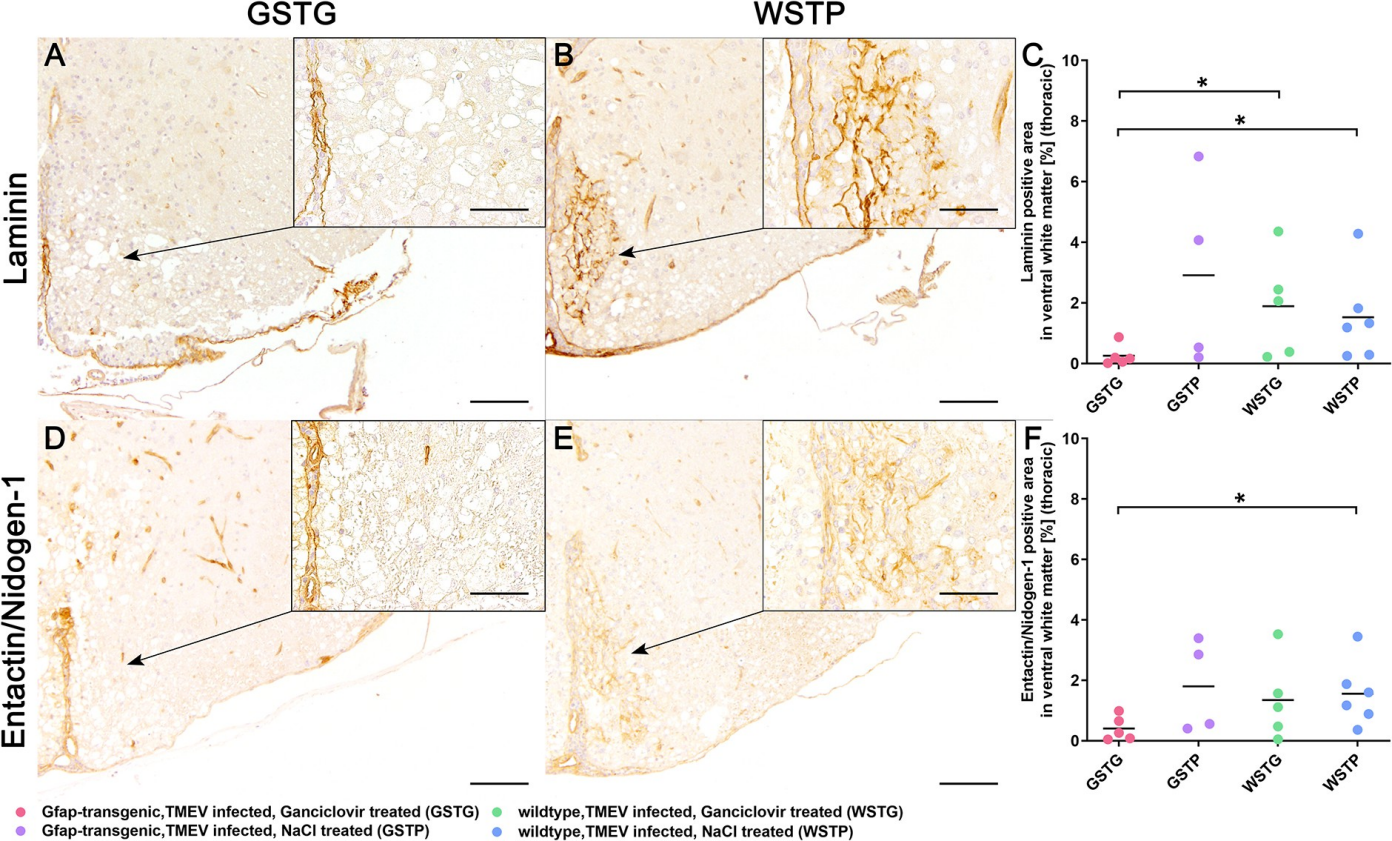
Impact of astrocyte depletion upon basement membrane extracellular matrix (ECM) components. Identification and quantification of basement ECM molecules was performed using immunohistochemistry identifying laminin (**A-C**) and entactin/nidogen-1 (**D-F**). Statistical analysis revealed a significant reduction of laminin and entactin/nidogen-1 in the lesioned ventral part of the thoracic spinal cord white matter in TMEV infected, ganciclovir treated, GFAP-transgenic (GSTG) mice at 77 dpi (**A, D**) compared to the non-astrocyte depleted control groups (**B, E**). Inserts visualize in more detail the increased intralesional accumulation of laminin (**B**) and entactin/nidogen-1 (**E**) in TMEV infected, natrium chloride (NaCl) treated wildtype (WSTP) animals compared to astrocyte depleted mice (**A**, **D**). For each antibody, one cross section of the thoracic spinal cord was evaluated per animal. Data are shown as scatter dot plots. The horizontal bar indicates the mean. Significant differences between GSTG and the control groups obtained by Kruskal-Wallis test followed by Mann–Whitney U post hoc tests are indicated by *, p ≤ 0.05. Bars represent 100 μm in overviews and 50 μm in the inserts.

Statistical analysis revealed a significant reduction of laminin in the thoracic spinal cord segment of GSTG mice compared to WSTG and WSTP control animals at 77 dpi (Fig 4A–4C).

As laminin is directly linked to entactin/nidogen-1 and both glycoproteins resemble major components of the basement membranes, the expression of entactin/nidogen-1 was investigated (Fig 4D–4F) [48–50]. At 77 dpi a reduction of entactin/nidogen-1 in the ventral thoracic spinal cord white matter of GSTG mice was detected compared to WSTP controls (Fig 4F).

### Quantification and subcellular localization of Kir 4.1 potassium channels

**Fig 5:**
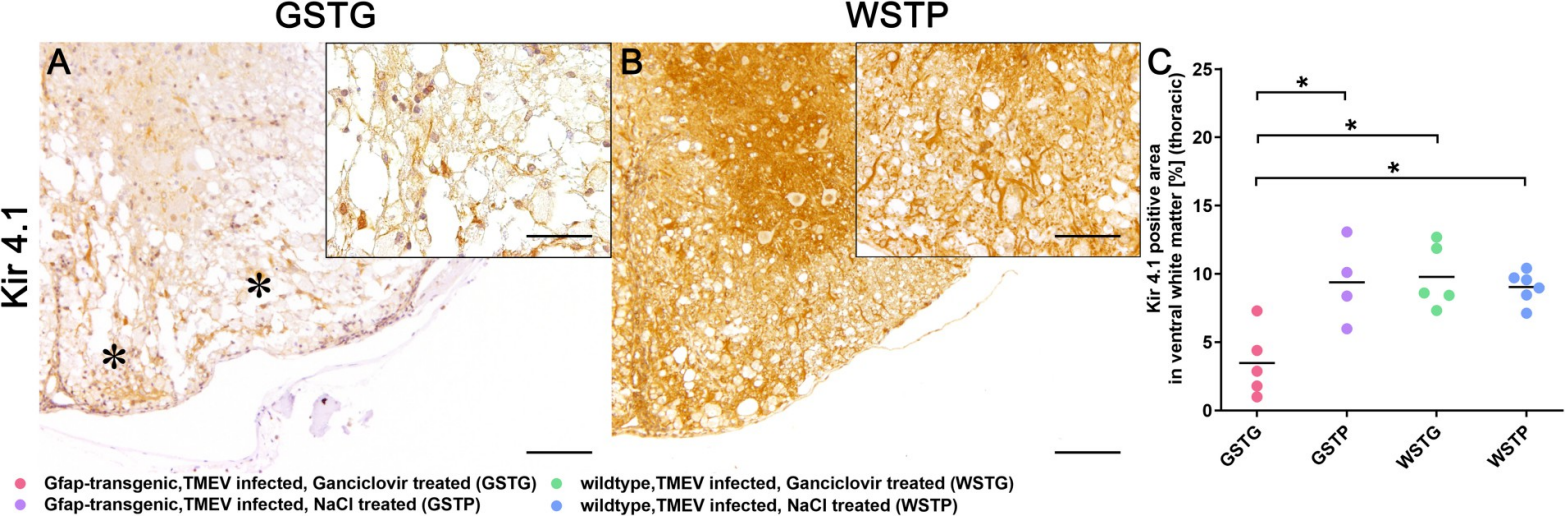
Effects of astrocyte depletion upon Kir 4.1 potassium channels. Immunohistochemistry targeting Kir 4.1 potassium channels revealed a significant reduction of Kir 4.1 positive area (asterisks, **A**) in the thoracic spinal cord of TMEV infected, ganciclovir treated, GFAP-transgenic (GSTG) mice compared to control animals (**B, C**). Inserts visualize in more detail the intralesional reduction of Kir 4.1 in astrocyte depleted animals (**A**) compared to the WSTP control group (**B**). For each antibody, one cross section of the thoracic spinal cord was evaluated per animal. Data are shown as scatter dot plots. The horizontal bar indicates the mean. Significant differences between GSTG and the control groups obtained by Kruskal-Wallis test followed by Mann–Whitney U post hoc tests are indicated by *, p ≤ 0.05. Bars represent 100 μm in overviews and 50 μm in the insert.

Immunohistochemistry targeting Kir 4.1 was applied to further evaluate the effects of the observed ECM molecule loss upon Kir 4.1 potassium channels (Fig 5A–5C). Statistical analysis revealed a significant reduction of potassium channels in the lesioned ventral parts of the thoracic spinal cord white matter of GSTG mice compared to TMEV infected, NaCl treated, GFAP-transgenic mice (GSTP), WSTG, and WSTP control animals (Fig 5C).

### Quantification of non-basement membrane ECM molecules

**Fig 6:**
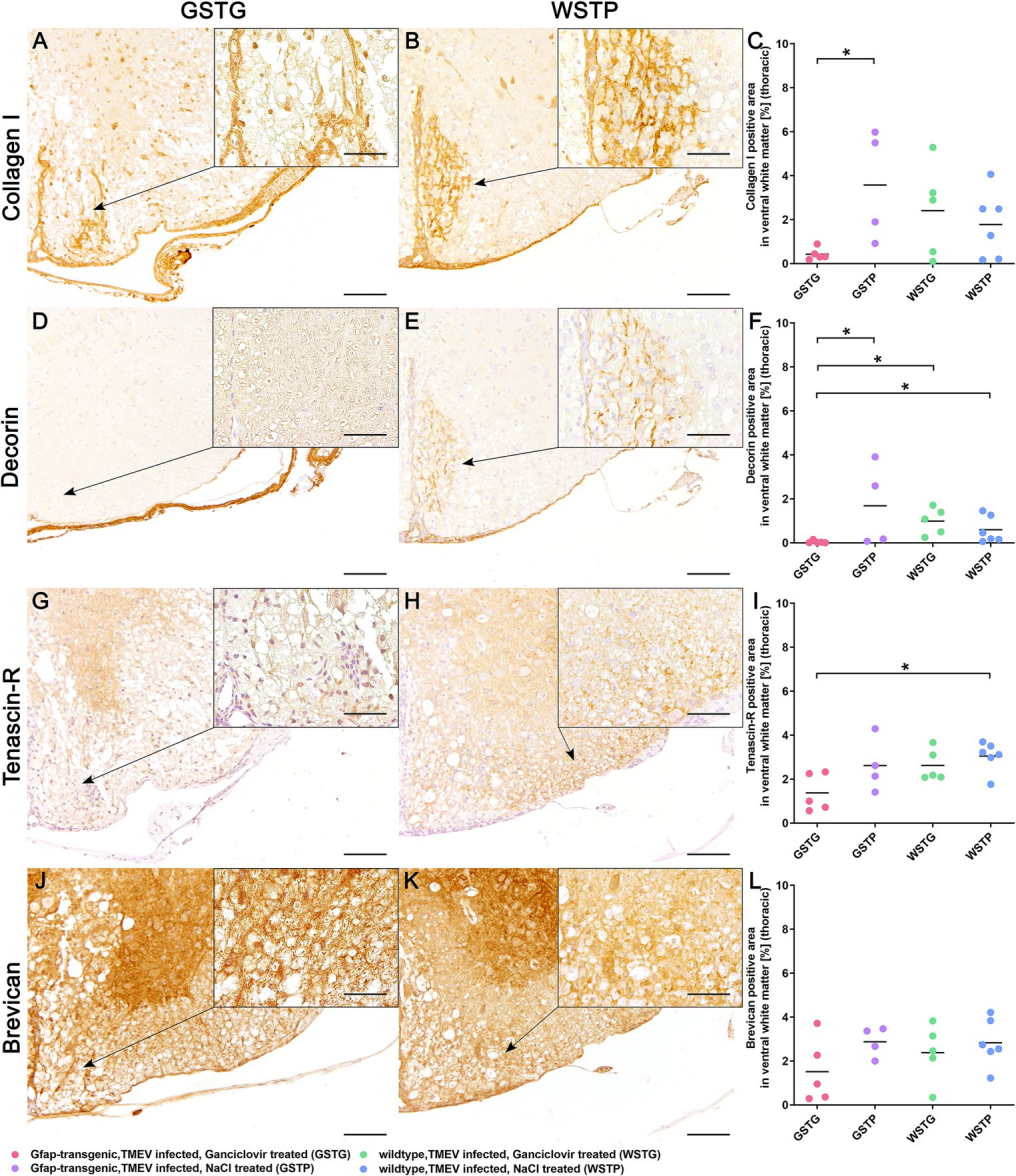
Impact of astrocyte depletion upon non-basement membrane extracellular matrix (ECM) components. Identification and quantification of basement ECM accumulation in the thoracic spinal cord white matter was performed using immunohistochemistry identifying collagen I (**A-C**), decorin (**D-F**), tenascin-R (**G-I**), and brevican (**J-L**). Statistical analysis revealed a significant reduction of collagen I in the ventral part of the thoracic spinal cord white matter in TMEV infected, ganciclovir treated, GFAP-transgenic (GSTG) mice (**A**) compared to TMEV infected, NaCl treated, GFAP-transgenic (GSTP) controls (**C**). Immunohistochemistry detecting decorin showed a significant decrease of decorin positive area in astrocyte depleted animals compared to their controls (**F**). Inserts visualize in more detail the increased intralesional accumulation of collagen I (**B**) and decorin (**E**) in WSTP control animals compared to astrocyte depleted mice (**A**, **D**). Furthermore, tenascin-R was significantly reduced in GSTG mice compared to TMEV infected, NaCl treated, wildtype (WSTP) controls (**I**). Loss of tenascin-R in GSTG animals was intralesionally observed in the thoracic spinal cord white matter as indicated by the arrow in **G**, whereas control animals showed a diffuse circumferential expression of tenascin-R as visualized in **H** and in more detail in the inserts (**G**, **H**). However, immunohistochemistry targeting brevican did not reveal a significant difference between GSTG mice (**J**) and the control animals (**K, L**), as shown also in higher magnification in the inserts (**J**, **K**). For each antibody, one cross section of the thoracic spinal cord was evaluated per animal. Data are shown as scatter dot plots. The horizontal bar indicates the mean. Significant differences between GSTG and the control groups obtained by Kruskal-Wallis test followed by Mann–Whitney U post hoc tests are indicated by *, p ≤ 0.05. Bars represent 100 μm in overviews and 50 μm in the inserts.

As astrocytes are involved in remodeling and maintenance of neuronal function and plasticity, immunohistochemistry targeting astrocyte associated, non-basement membrane ECM molecules such as collagen I, decorin, tenascin-R, and brevican was applied [2, 35]. Previous studies reported a diffuse and intralesional accumulation of collagen I after CNS insults [13, 51]. Collagen I expression was detected with a variable degree in all TMEV infected animals (Fig 6A–6C). Additionally, GSTG mice showed a significant reduction of collagen I positive area compared to their NaCl treated control group (Fig 6C, S1 Fig).

During CNS injury the, non-basement membrane ECM molecule decorin is upregulated by astrocytes and endothelial cells promoting axonal growth, reducing inflammatory reactions and stimulating angiogenesis [52–54]. In the present study astrocyte depleted animals showed a significant reduction of decorin compared to their controls GSTP, WSTG, and WSTP (Fig 6D–6F).

Furthermore the amount of the non-basement membrane ECM molecule tenascin-R was investigated using immunohistochemistry (Fig 6G–6I). GSTG mice showed a significant reduction of the tenascin-R positive area in the lesioned ventral thoracic spinal cord white matter compared to WSTP mice at 77 dpi (Fig 6I).

Previous publications reported an interaction between tenascin-R and brevican [36]. To investigate, whether co-regulatory processes for tenascin-R and brevican exist after astrocyte depletion, brevican was visualized by immunohistochemistry (Fig 6J–6L). However, morphometric quantification of the brevican immunolabeled areas revealed no significant differences between all groups (Fig 6L).

### Effects of astrocyte depletion upon perineuronal nets integrity

**Fig 7:**
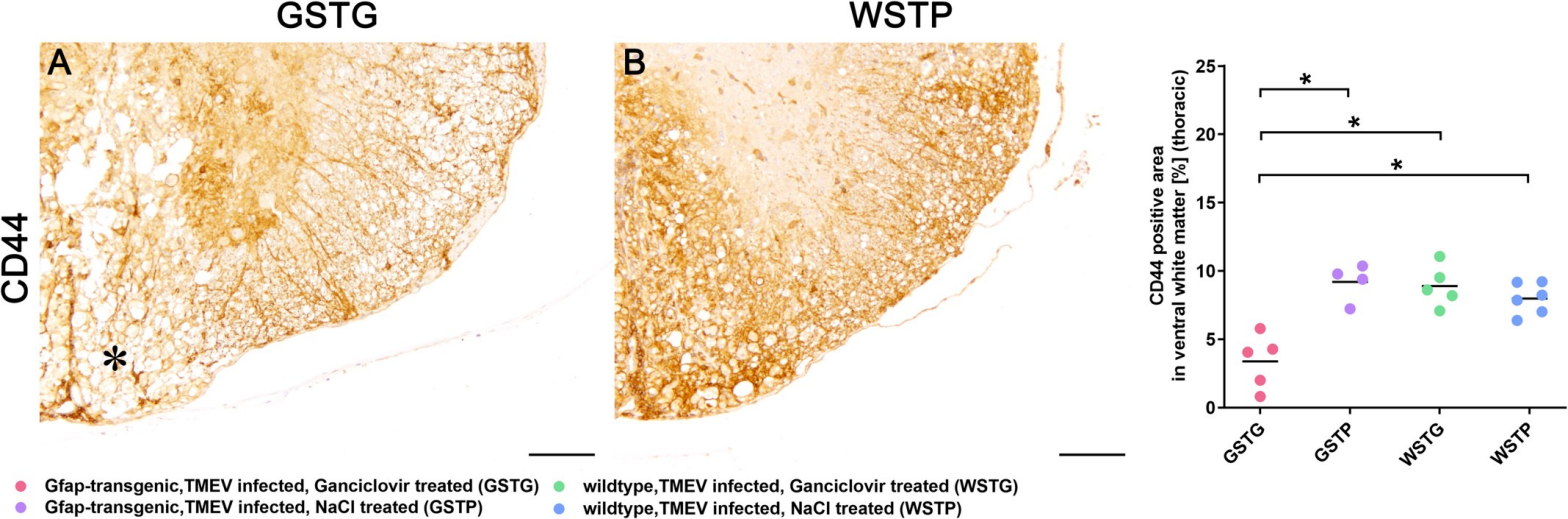
CD44 expression during astrocyte depletion. Immunohistochemistry targeting the cell-surface glycoprotein CD44 was used to evaluate the anchoring of extracellular matrix to astrocytes. Statistical analysis revealed a significant reduction of CD44 positive area in TMEV infected, ganciclovir treated, GFAP-transgenic (GSTG) mice compared to all control groups (**A**-**C**). A reduction of the CD44 labeled area in the ventral spinal cord white matter of GSTG animals (asterisk, **A**) compared to a diffuse and circumferential immunolabelling in control animals (**B**, **C**) was detected. For each antibody, one cross section of the thoracic spinal cord was evaluated per animal. Data are shown as scatter dot plots. The horizontal bar indicates the mean. Significant differences between GSTG and the control groups obtained by Kruskal-Wallis test followed by Mann–Whitney U post hoc tests are indicated by *, p ≤ 0.05. Bars represent 100 μm.

Using a hyaluronan backbone, non-basement membrane ECM molecules like tenascin-R and brevican are anchored to astrocyte foot processes by the cell-surface glycoprotein CD44 contributing to the integrity of perineuronal nets [36, 37]. Therefore, immunohistochemistry targeting CD44 was performed to evaluate the effect of astrocyte depletion upon cell-surface glycoprotein expression and to investigate the effect of a possible aberrant CD44 expression together with the observed loss of tenascin-R on PNN integrity (Fig 7A–7C). At 77 dpi GSTG mice revealed a significant reduction of CD44 within the ventral parts of the thoracic spinal cord white matter compared to all control groups (Fig 7C).

## Discussion

Progression of demyelinating diseases is frequently accompanied by deterioration of clinical signs in human diseases like multiple sclerosis and its animal models [6, 55]. Haist *et al*. (2012) demonstrated an association between clinical deterioration and deposition of ECM components during TMEV-IDD. The deposition of astrocyte derived ECM glycoproteins in the CNS plays a crucial role at later stages of demyelinating and inflammatory diseases of the CNS such as multiple sclerosis [13]. During disease progression intralesional alterations of ECM molecule receptors may occur, resulting in a detachment of ECM components like laminin from the vascular basement membrane, leading to a disruption of the blood-brain barrier [56]. Furthermore, an involvement of ECM molecules in signal transduction to inflammatory and glial cells as well as neurons in MS lesions is suggested [57]. Therefore, further studies investigating the role of ECM molecules to the progression of demyelinating diseases in established animal models like TME are needed.

In a recent study, we evaluated the impact of astrocyte depletion upon clinical manifestation, inflammation and demyelination using TMEV-IDD as a virus-induced animal model for multiple sclerosis. However, the mechanisms leading to clinical deterioration remained elusive and an altered composition of the ECM was postulated to contribute to the clinical deterioration [4]. Therefore, the aim of the present study was to evaluate the consequences of astrocyte depletion upon the composition of ECM molecules of the basement membrane and tripartide synapse.

In the present study, a significant reduction of ECM glycoproteins in the thoracic spinal cords of GFAP knockout compared to control mice was identified using azan and picrosirius red stainings (Fig 3). These data indicate that depletion of astrocytes significantly reduces the deposition of ECM glycoproteins in the spinal cord underlining the importance of astrocytes as major contributors to ECM deposition. To further specify the results of the histochemical investigations, representative astrocyte associated basement and non-basement ECM molecules were selected and quantified. Within the lipid membrane, the basement membrane ECM molecule laminin is linked to the dystroglycan-dystrophin-dystrobrevin complex ensuring the cell-surface expression of water channels like AQP4. Previous studies showed a coexpression of laminin and AQP4 on the cell surface by culturing astrocytes in the presence of laminin [58, 59]. In the present study, depletion of astrocytes led to a significant reduction of laminin in the lesioned thoracic spinal cord white matter in GFAP knockout mice (Fig 4A–4C). This result complements the observations of our previous study very well. Here, a reduced AQP4 expression was accompanied by a rounded shape and limited number of astrocytic processes [4]. Thus, it can be assumed, that the loss of laminin during astrocyte depletion may have induced a conformational change in the laminin-dystroglycan-syntrophin-AQP4 complex resulting in a loss of AQP4, reduced process formation, and disrupted basement membrane integrity.

Assembly and organization of the basement membrane is not restricted to the presence of laminin, but also requires direct linkage to entactin/nidogen-1 for assurance of basement membrane integrity [60]. Therefore, the reduced laminin expression observed in this study may have contributed to a significant reduction of entactin/nidogen-1, further underlining an impaired basement membrane integrity (Fig 4D–4F).

Different studies already postulated a co-expression of Kir 4.1 and AQP4 in a laminin dependent manner [59, 61–63]. In the present study a significant loss of the Kir 4.1 potassium channels in astrocyte depleted mice was detected (Fig 5) along with the laminin reduction. Since Kir 4.1 and AQP4 are particularly enriched in the plasma membrane close to blood vessels, a direct correlation of potassium buffering and water flux is assumed [61, 63]. Accordingly, loss of Kir 4.1 and reduced expression of AQP4 after astrocyte depletion may have led to a disrupted neuroprotective microenvironment resulting in the clinical deterioration of TMEV infected animals.

During CNS injury, activation of astrocytes leads to an upregulation of ECM glycoprotein secretion and intralesional ECM accumulation [13]. Ultimately, this results in the formation of a glial scar and reduced inflammatory cell invasion [13]. In the present study, the non-basement ECM molecules collagen I and decorin were intralesionally detected in all TMEV infected animals (Fig 6A–6F). Furthermore, GFAP knockout mice showed a significant reduction of these ECM components. The reduction of scar forming ECM molecules did not lead to an increased but a decreased number of inflammatory cells in the CNS. This phenomenon may be explained by the fact that recruitment and retention of T lymphocytes is dependent on their adhesion to ECM molecules [64]. However, the reduced number of inflammatory cells is unlikely to have contributed to the clinical deterioration of the astrocyte depleted animals. Therefore, other factors like a reduced activation and differentiation of OPCs as well as a neuronal dysfunction without morphological correlate may have contributed to the clinical deterioration of the animals.

Astrocytes are not only participating in formation of the BSCB, they also play a crucial role in formation of PNNs [44]. Thereby, astrocyte associated non-basement membrane ECM molecules form PNNs for preservation of synapse plasticity. However, the complex underlying mechanisms remain incompletely understood [65]. Previous studies reported an involvement of brevican and tenascin-R in the long-term potentiation of the murine CNS [66–68]. While the importance of tenascin-R for synaptic plasticity and function was demonstrated in murine animal models, brevican is less important for structure and function in the CNS [66–68]. Tenascin-R knockout mice display an impairment of motor coordination and increased anxiety levels, while brevican knockout mice did not exhibit deficits in learning and memory [68, 69]. In the present study, tenascin-R was significantly downregulated in the thoracic spinal cord of TMEV-infected GFAP knockout compared to wildtype control mice, whereas statistical analysis did not detect a significant difference in brevican expression levels (Fig 6G–6L). Although the animals displayed a deterioration of the motor coordination, the amount of axonal damage was not increased, as previously shown using immunohistochemistry targeting non-phosphorylated-neurofilaments [4]. Therefore, it can be assumed, that the downregulated ECM glycoprotein tenascin-R plays an important role in the synaptic function of neurons as well as saltatory conduction in the murine spinal cord as described for the murine dentate gyrus [70]. As already suggested in a previous study our results also indicate, that tenascin-R seems to be more important for predicting the clinical outcome than brevican.

CD44 represents another indispensable glycoprotein sustaining the integrity of tripartide synapses and PNNs [40]. The contribution of CD44 to demyelinating disease progression was previously investigated in other animal models for MS like experimental autoimmune encephalomyelitis [EAE; 71]. CD44 knock out during EAE led to an increased severity of disease associated with an increase of inflammation and a proinflammatory cytokine environment [71]. In the present study, deterioration of clinical signs during TMEV infection was accompanied by a significant reduction of CD44 positive area in the lesioned ventral part of astrocyte depleted mice compared to all control groups (Fig 7).

According to previous studies, CD44 is critically involved in synaptic excitatory transmission as well as functional and structural plasticity of dendritic spines [72]. Therefore, the clinical deterioration of GFAP depleted mice in the present study may be partially attributed to a substantial loss of CD44 that ultimately led, together with a loss of the CD44-anchored proteoglycan tenascin-R, to structural alteration of the PNNs and consequent limitation of synaptic function. However, it cannot be ruled out that a downregulation of CD44 in other cell types like neurons or OPCs may have also contribute to the deterioration of clinical signs. In summary, the present study showed, that astrocytes play a key role in production of basement membrane and non-basement membrane ECM molecules in the inflamed and demyelinated spinal cord. Subsequently, depletion of astrocytes was associated with a significant reduction of these molecules. Whether the deterioration of clinical signs was directly or indirectly induced by the deposition of ECM components remains still elusive. The loss of Kir 4.1 potassium channels together with a reduced expression of AQP4 may have resulted in an impaired potassium buffering and disrupted fluid transport significantly altering the neuroprotective milieu. Furthermore, loss of the glycoprotein CD44 may have disrupted the integrity of PNNs, directly affecting neuronal function and plasticity and finally resulting in a deterioration of clinical signs.

## Supporting information

S1 Fig Impact of astrocyte depletion upon collagen I. Identification and quantification of collagen I accumulation in the thoracic spinal cord white matter was performed using immunohistochemistry. Statistical analysis revealed a significant reduction of collagen I in the lesioned thoracic spinal cord white matter in TMEV infected, ganciclovir treated, GFAP-transgenic (GSTG) mice (A) compared to TMEV infected, NaCl treated, GFAP-transgenic (GSTP) controls (B). Inserts visualize in more detail the intralesional accumulation of collagen I. Bars represent 100 μm in overviews and 50 μm in the inserts.
(TIF)
