# Supplementary figures and images for: Astrocyte depletion alters extracellular matrix composition in the demyelinating phase of Theiler’s murine encephalomyelitis
Source: PLoS One. 2022 Jun 17;17(6):e0270239. doi: 10.1371/journal.pone.0270239 (PMC9205503; doi:10.1371/journal.pone.0270239)

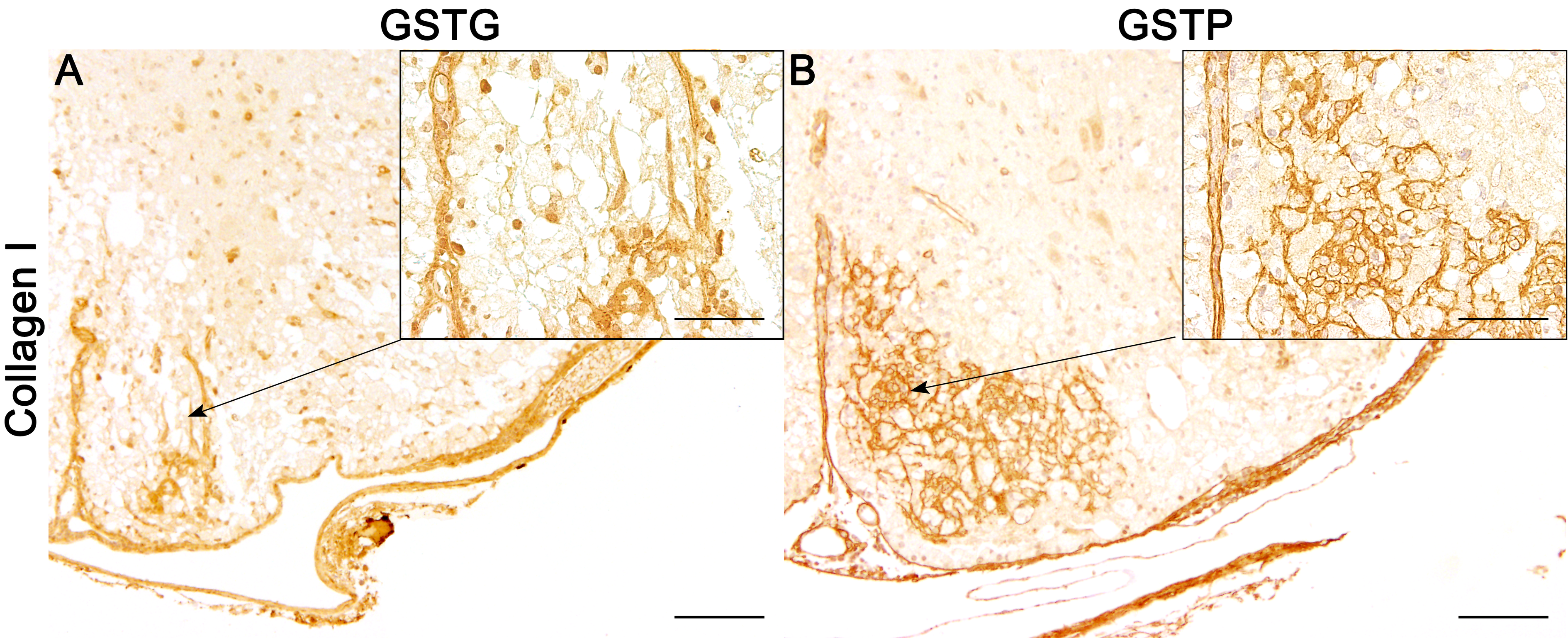

Supplement: S1 Fig — Identification and quantification of collagen I accumulation in the thoracic spinal cord white matter was performed using immunohistochemistry. Statistical analysis revealed a significant reduction of collagen I in the lesioned thoracic spinal cord white matter in TMEV infected, ganciclovir treated, GFAP-transgenic (GSTG) mice (A) compared to TMEV infected, NaCl treated, GFAP-transgenic (GSTP) controls (B). Inserts visualize in more detail the intralesional accumulation of collagen I. Bars represent 100 μm in overviews and 50 μm in the inserts. (TIF) [file pone.0270239.s001.tif]
